# Performance of the Fracture Risk Assessment Tool Associated with Muscle Mass Measurements and Handgrip to Screen for the Risk of Osteoporosis in Young Postmenopausal Women

**DOI:** 10.1055/s-0041-1741408

**Published:** 2022-01-29

**Authors:** Marina Martinelli Sonnenfeld, Fernanda Lellis Pimentel, Elizabeth Jeha Nasser, Luciano de Melo Pompei, Cesar Eduardo Fernandes, Marcelo Luis Steiner

**Affiliations:** 1 Gynecology and Obstetrics Department, Centro Universitário FMABC, Santo André, SP, Brazil

**Keywords:** osteoporosis, menopause, FRAX, NOGG, sarcopenia, osteoporose, menopausa, FRAX, NOGG, sarcopenia

## Abstract

**Objective** To evaluate the improvement in screening accuracy of the Fracture Risk Assessment Tool (FRAX) for the risk of developing osteoporosis among young postmenopausal women by associating with it clinical muscle mass measures.

**Methods** A sample of postmenopausal women was submitted to calcaneal quantitative ultrasound (QUS), application of the FRAX questionnaire, and screening for the risk of developing sarcopenia at a health fair held in the city of São Bernardo do Campo in 2019. The sample also underwent anthropometric measurements, muscle mass, walking speed and handgrip tests. A major osteoporotic fracture (MOF) risk ≥ 8.5% on the FRAX, a classification of medium risk on the clinical guideline of the National Osteoporosis Guideline Group (NOGG), and a QUS T-score ≤ -1.8 sd were considered risks of having low bone mass, and QUS T-score ≤ -2.5sd, risk of having fractures.

**Results** In total, 198 women were evaluated, with a median age of 64 ±  7.7 years, median body mass index (BMI) of 27.3 ±  5.3 kg/m ^2^ and median QUS T-score of −1.3 ±  1.3 sd. The accuracy of the FRAX with a MOF risk ≥ 8.5% to identify women with T-scores ≤ -1.8 sd was poor, with an area under the curve (AUC) of 0.604 (95% confidence interval [95%CI]: 0.509–0.694) for women under 65 years of age, and of 0.642 (95%CI: 0.571–0.709) when age was not considered. Including data on muscle mass in the statistical analysis led to a significant improvement for the group of women under 65 years of age, with an AUC of 0,705 (95%CI: 0.612–0.786). The ability of the high-risk NOGG tool to identify T-scores ≤ -1.8 sd was limited.

**Conclusion** Clinical muscle mass measurements increased the accuracy of the FRAX to screen for osteoporosis in women aged under 65 years.

## Resumo

**Objetivo** Avaliar a melhora da precisão da Fracture Risk Assessment Tool (Ferramenta de Avaliação do Risco de Fraturas, FRAX, em inglês) no rastreio do risco de desenvolver osteoporose em mulheres jovens pós-menopáusicas com a associação de medidas clínicas de massa muscular e preensão manual.

**Métodos** Uma amostra de mulheres pós-menopáusicas foi submetida a ultrassom quantitativo (USQ) de calcâneo, à aplicação do questionário FRAX, e rastreadas quanto ao risco de desenvolver sarcopenia em uma feira de saúde realizada em 2019 em São Bernardo do Campo. Além disso, a amostra também foi submetida a antropometria, e a testes de massa muscular, velocidade de marcha, e preensão manual. Um risco de grandes fraturas osteoporóticas (GFOs) ≥ 8,5% no FRAX, classificação de médio risco nas diretrizes clínicas do National Osteoporosis Guideline Group (NOGG), e *T-score* no USQ ≤ -1,8 dp foram considerados riscos de ter baixa massa óssea, e *T-score* no QUS ≤ -2,5 sd, risco de ter fraturas.

**Resultados** Ao todo, 198 mulheres foram avaliadas, com idade média de 64 ±  7,7 anos, índice de massa corporal (IMC) médio de 27,3 ±  5,3 kg/m ^2^ , e *T-score* médio no USQ de -1,3 ±  1,3 sd. A precisão do FRAX com um risco de GFO ≥ 8,5% para identificar mulheres com *T-score* ≤ -1,8 dp foi precária, com uma área sob a curva (ASC) de 0,604 (intervalo de confiança de 95% [IC95%]: 0,509–0,694), para mulheres menores de 65 anos de idade, e de 0,642 (IC95%: 0,571–0,709) quando a idade não foi considerada. A inclusão de dados da massa muscular na análise estatística levou a uma melhora significativa no grupo menor de 65 anos de idade, com uma ASC de 0,705 (IC95%: 0,612–0,786). A habilidade da ferramenta NOGG de alto risco para identificar *T-scores* ≤ -1,8 dp foi limitada.

**Conclusão** As medidas clínicas da massa muscular aumentaram a precisão do FRAX no rastreio de osteoporose em mulheres menores de 65 anos de idade.

## Introduction

An increase in life expectancy and an aging population are associated with a higher prevalence of osteoporosis and fragility fractures. This certainly occurs in Brazil, where life expectancy has increased from 50 years in 1952 to 71 in 2010, and is estimated to be 80 years by 2050. 1 2

One of the main challenges in osteoporosis care is the identification of individuals at a higher risk of incurring in fractures and, accordingly, the establishment of a preventive therapeutic approach. In last few years, clinical tools, associated or not to dual-energy X-ray absorptiometry (DXA), have been developed to improve the accuracy of fracture identification. The use of calcaneal quantitative ultrasound (QUS), a method more practical and less expensive than DXA, to predict the risk of fracture is also recommended. According to the World Health Organization (WHO), QUS cannot be used to diagnose osteoporosis or to monitor the effectiveness of the therapy. There are, however, studies 3 4 that confirm that QUS can predict fractures in elderly women, such as the one by Moayyeri et al. 3 (2012) a meta-analysis with a total follow-up of 279,124 people.

Clinical tools such as the Garvan fracture risk calculator, the QFracture risk calculator, and the Fracture Risk Assessment Tool (FRAX; https://www.sheffield.ac.uk/FRAX/ ) combine age and gender with clinical risk factors to estimate the risk of fracture in the next 5 or 10 years. There are also tools for osteoporosis screening, mainly for women younger than 65 years of age, as DXA is not universally recommended. Tools such as the Simple Calculated Osteoporosis Risk Estimate (SCORE), the Osteoporosis Self-Assessment Tool (OST), and the Osteoporosis Risk Assessment Instrument (ORAI), and even the FRAX, may be mentioned, as there is no standard for the analysis of this population. 5 6

Brazilian guidelines recommend the use of the FRAX associated to the strategy for screening of the National Osteoporosis Guideline Group (NOGG), which enables the classification of individuals into high-, medium- and low-risk groups for fragility fractures. Those in the high-risk group should receive pharmacological treatment, those in the medium-risk group should undergo DXA as screening for osteoporosis, and those in the low-risk group should be advised on their lifestyle habits. 7 8

Although these strategies are recommended, very few studies 9 have evaluated their accuracy in identifying the risk of fracture and in tracking osteoporosis in the Brazilian population. In the young American postmenopausal population, the performance of the FRAX in identifying women with a risk of incurring in fractures was poor. 12 Simpler tools than the FRAX, such as the OST, have shown a better specificity, but they also demonstrate low sensitivity. 9 10 11 12

The progressive loss of skeletal muscle mass and function in conjunction with aging is known as sarcopenia. It is considered a component of frailty syndrome leading to a higher risk of falling and fragility fractures. Its diagnosis is based on the assessment of muscle force and physical performance. The identification of individuals at risk of developing sarcopenia is simple, and it can be performed in ambulatory care. 13 14 15

Osteoporosis and sarcopenia are usually connected to one another and both contribute to disability and frailty in the elderly. Nevertheless, clinical signs of sarcopenia or muscular mass evaluations are not incorporated in the clinical tools for the assessment of the risk of fracture. 15

Therefore, the present study aims to evaluate the performance of the FRAX associated with skeletal muscular mass analyses in screening and diagnosing postmenopausal osteoporosis.

## Methods

### Population

In the present cross-sectional study, clinical data and supplementary exams were reviewed, after they were collected during the XXII Maratona da Saúde e Cidadania Dr. Claudio Zago, a health fair health held on April 13th, 2019, by the São Bernardo do Campo Rotary Club. In this event, the department of obstetrics and gynecology of Faculdade de Medicina do ABC (FMABC) invited postmenopausal women aged 50 years or older to take part in the activities in their booth. The activities comprised the application of structured clinical questionnaires on sarcopenia and osteoporosis, assessments of the height, weight, and circumferences of the arm, thigh, and calf, a walking speed test, the handgrip strength test, and the performance of a calcaneal QUS. The present study was approved by the Ethics in Research Committee of FMABC.

### Procedures

#### Questionnaires

The subjects answered three specific questionnaires: the first one involved personal and clinical data, the second one was regarding the risk of bone fracture in 10 years (FRAX), and the third one was on the risk of developing sarcopenia. All questionnaires were applied by trained medicine students.

#### Clinical Questionnaire

The subjects were asked about their age, weight, height, ethnicity, time since the onset of menopause, previous use of hormone replacement therapy, smoking and/or drinking habits, the regularity of physical activity and muscle mass performance.

#### Sarcopenia Questionnaire

The subjects answered the Strength, assistance with walking, rising from a chair, climbing stairs, and falls (SARC-F) questionnaire. Developed by American researchers, it identifies people with increased risk of developing sarcopenia through five questions approaching the areas in its name: strength, assistance with walking, rising from a chair, climbing stairs, and falls. Each answer is scored from 0 to 2, resulting in a final score ranging from 0 to 10. Scores ≥ 6 indicate a higher risk of developing sarcopenia. 14 16

#### FRAX Questionnaire

All subjects were submitted to the FRAX-Brazil quationnaire. This clinical tool developed by the WHO matches clinical data and estimates the percentage risk of hip fracture and major fractures (clinical spinal, forearm, hip and shoulder fractures) for the following 10 years. In the present study, a risk of major osteoporotic fracture (MOF) ≥ 8.5% on the FRAX was adopted as the criteria to perform a supplementary bone densitometry exam. 17

#### NOGG Grading

Using the NOGG tool (available at https://www.sheffield.ac.uk/NOGG/ ), the subjects were classified in low-, medium- and high-risk groups. The NOGG tool recommends that people in the medium-risk group should undergo the bone density test to screen for osteoporosis. In the present study, we chose to group the individuals classified as medium- and high-risk according to the NOGG tool, considering that this is the population for whom densiometry should be requested or who should undergo pharmacological treatment.

#### Anthropometric and Muscle Mass Measurements

The measurements of height, weight and of the circumferences of the arm, thigh and calf were made with a measuring tape and a Geratherm scale. During weighing, the patients were guided to take off their coats and bags. The measurment of the circumferences was standardized as follows: 13 17 18 19 20 21 22 23 24 25 26 27 28

Arm – midpoint between the lateral projection of the acromion process of the scapula and the lower margin of the ulnar olecranon.

Calf – at its widest point.

Thigh – midpoint on the trochanteric and the margin of the kneecap.

#### Assessment of Sarcopenia

The subjects were assessed according to the definition of sarcopenia of the European Working Group on Sarcopenia in Older People (EWGSOP). 14 16 To calculate the muscle mass (MM) in kilos (Kg) recomended by the EWGSOP, the predictive equation described by Lee et al. 18 (2000) was used, in which:

- MM (Kg) = Ht x (0.00744 x AC ^2^ + 0.00088 x TC ^2^ + 0.00441 x CC ^2^ ) + 2.4 x gender – 0.048 × age + race + 7,8.

In the equation, Ht refers to the height in centimeters (cm), AC, to the arm circumference in cm, TC, to the thigh circumference in cm, and CC, to the calf circumference in cm. Regarding gender, the value of 1 is considered for men, and 0 for women; as for race, the values are -2.0 for Asians, 1.1 for African-Americans, and 0 for Whites or Hispanics. In the present study, the race score was adapted, considering -2.0 for Asians, 1.1 for people who considered themselves black or brown ( *pardo* or *negro* , in Portuguese) and 0 for people who considered themselves white ( *branco* , in Portuguese).

The skeletal MM index was calculated as MM divided by the squared height. Subjects with values between 5.5 kg/m ^2^ and 6.76 kg/m ^2^ were considered at risk of developing sarcopenia. The muscle strength was evaluated using an electronic handgrip, which estimates the person's muscle strength in kg based on the maximum strength reached in palm pressure. The measurements were recorded using the dominant arm, with the woman standing up straight, with both arms straight down and equidistant feet. The gadget was previously calibrated for females aged ∼ 60 years. Women with results bellow 20 kg were considered at risk of developing sarcopenia. 19 20 21

Finally, a walking speed test was used. The women would walk a distance of 6 m, in which the first meter was used to increase the walking speed, the 4 following meters were for timing the normal walking speed, and the last meter, for deceleration. Those with a time ≥ 0.8m/s were considered at risk of developing sarcopenia.

#### Calcaneal Quantitative Ultrasound (QUS)

All subjects underwent calcaneal QUS, with the GE Lunar Achilles Express ultrasonometer (GE Healthcare, Chicasgo, IL, US), through which the standard deviation values of bone mass related to the young adult population (T-score) can be obtained, as well as those with the same age (Z-score). In the present study, subjects with T-scores ≤ -1.8 sd were considered at risk of developing osteoporosis, and those with scores ≤ -2.5 sd, at risk of incurring in fractures. 29

### Statistical Analysis

The Microsoft Excel 2018 (Microsoft Corp. Redmond, WA, US), version 1910, was used to organize the data obtained, and the MedCalc Statistical Software (MedCalc Software bv, Ostend, Belgium), version 19.1, was used to conduct the statistical analysis. The Kolmogorov-Smirnov test was used to test the normal distribution of the numeric data. The continuous numeric data was expressed as means ±  standard deviations, and the categorical data, as frequencies and percentages. The comparison of the groups was performed using the Student *t* -test for independent samples when the continuous numeric data followed a normal distribution, and the Wilcoxon test, for the data which did not follow a normal distribution. For the categorical data, the comparisons were made using the Chi-squared test. The diagnostic accuracy was evaluated through the area under the curve (AUC), following the methodology described by DeLong et al. 30 In all scenarios, a level of significance of 5% was adopted.

## Results

A total of 200 patients were evaluated, 2 of whom were excluded for having weight higher than that allowed by the FRAX. The median age was of 64 ±  7.7 years, the median body mass index (BMI) was of 27.3 ±  5.3 kg/m ^2^ , and the median T-score in the QUS was od -1.3 sd, ( Table 1 ). In the comparison of age groups, the population aged ≥ 65 years obtained inferior values, which was statistically significant, in the parameters related to fat, lean and bone mass, as well as in the SARC-F and physical performance ( Table 1 ).

**Table 1:** Clinical and anthropometric characteristics, diagnostic parameters of sarcopenia, and bone density of the study sample and comparison when divided by age group

	TOTAL	AGE			
		≤ 65 years ( *N* = 115)	> 65 years ( *N* = 83)	*p* *	
	**Mean ± standard deviation**	**Mean ± standard deviation**	**Mean ± standard deviation**		
Weight (Kg)	65.8 ± 13.1	67.6 ± 14.1	63.8 ± 10.8	0.0170	
Height (m)	1.5 ± 0.1	1.55 ± 0.1	1.5 ± 0.1	< 0.0001	
BMI (Kg/m ^2^ )	27.3 ± 5.3	27.7 ± 5.8	26.8 ± 4.4	< 0.0001	
AC (cm)	30 ± 4.4	30 ± 4.3	29 ± 4.4	< 0.0001	
TC (cm)	50 ± 6.3	51 ± 6.1	49.5 ± 6.5	< 0.0001	
CC (cm)	36 ± 4.0	37 ± 4.0	35 ± 4.0	< 0.0001	
MM (Kg)	27.7 ± 5.9	28.8 ± 5.9	26.1 ± 5.2	< 0.0001	
SMMI (Kg/m ^2^ )	11.3 ± 2.3	11.8 ± 2.3	10.92 ± 2.0	< 0.0001	
Handgrip (Kg)	21 ± 5.5	22.3 ± 5.3	19.4 ± 5.5	< 0.0001	
GS (m/s)	0.89 ± 0.2	0.9 ± 0.2	0.85 ± 0.2	< 0.0001	
*T-score* on the calcaneal QUS	-1.3 ± 1.3	-1 ± 1.2	-1.6 ± 1.2	< 0.0001	
SARC-F	2 ± 2.3	1 ± 2.4	2 ± 2.2	< 0.0001	
		**n (%)**	**n (%)**	**n (%)**	***p*** **
Ethnicity	White	119 (60.1)	64 (55.7)	55 (66.3)	0.124
	Brown or Black	68 (34.3)	46 (40)	22 (26.5)	
	Asian	11 (5.5)	5 (4.3)	6 (7.2)	
Level of schooling	Illiterate	3 (1.5)	2 (1.7)	1 (1.2)	0.259
	Incomplete Primary Education	47 (23.7)	29 (25.2)	18 (21.7)	
	Complete Primary Education	31 (15.6)	15 (13.0)	16 (19.3)	
	Incomplete High School	72 (36.3)	47 (40.9)	25 (30.1)	
	Complete High School	16 (8.0)	10 (8.7)	6 (7.2)	
	Higher Education	29 (14.6)	12 (10.4)	17 (20.5)	
SARC-F ≥ 6	Yes	23 (11.6)	12 (10.4)	8 (9.6)	0.956
	No	174 (88.3)	103 (89.6)	75 (90.4)	
T-score ≤ -1.8 sd	Yes	58 (29.3)	23 (20)	35 (42.2)	0.001
	No	140 (70.7)	92 (80)	48 (57.9)	
Z-score ≤ -2.5 sd	Yes	27 (13.6%)	9 (7.8)	18 (21.7)	0.005
	No	171 (86.4%)	106 (92.2)	65 (78.3)	

; AC, arm circumference; BMI, body mass index, CC, calf circumference; MM, muscle mass; SMMI, skeletal muscle mass index; VM - gait speed; QUS, quantitative ultrasound; SARC-F, Strength, assistance with walking, rising from a chair, climbing stairs, and falls questionnaire; TC, thigh circumference.

Notes: *Correlation between age and the evaluated data through the Wilcoxon correlation test; **comparison between age over 65 years and ≤ 65 years by the Chi-squared test.

**Fig. 1:**
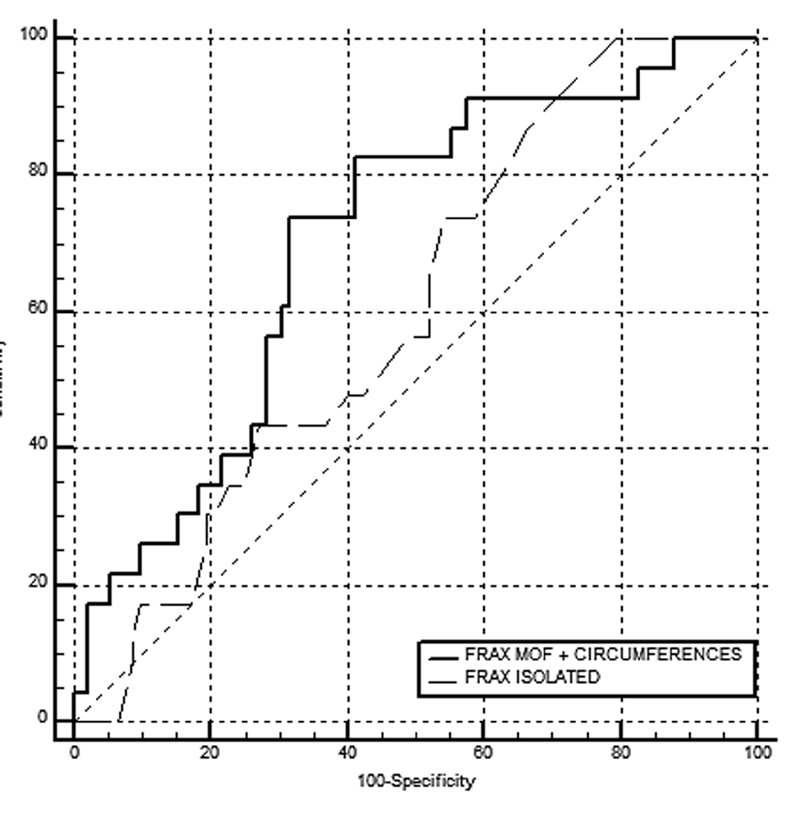
Performance of the Fracture Risk Assessment Tool (FRAX) regarding the risk of major osteoporotic fracture (MOF) taken in isolation and associated with circumference measurements to identify women under 65 years of age with T-score ≤ -1.8 sd on calcaneal quantitative ultrasound (QUS).

**Fig. 2:**
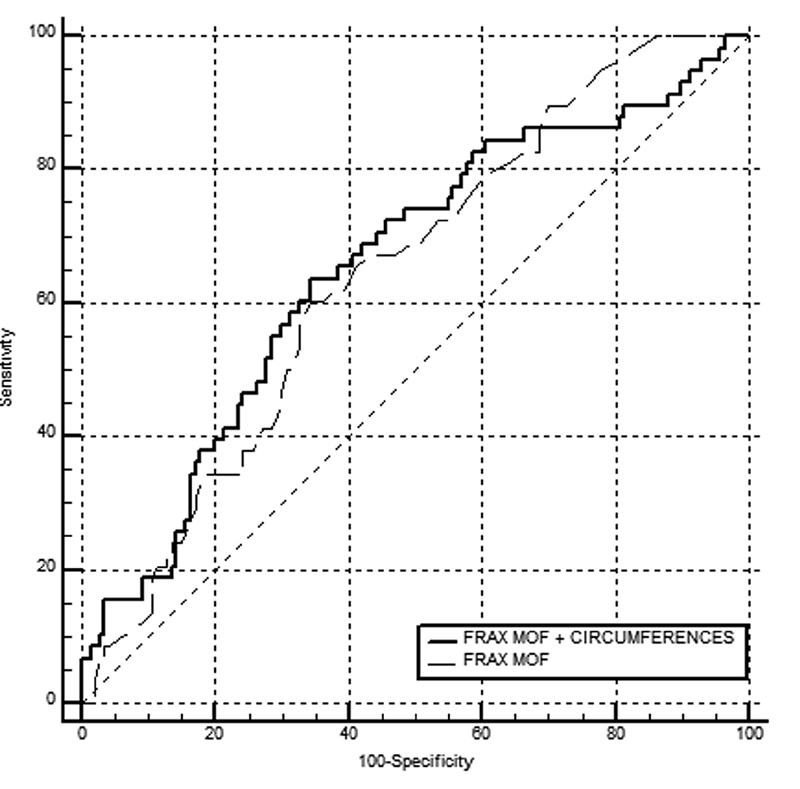
Performance of the FRAX regarding the MOF risk taken in isolation and associated with circumference measurements to identify women with a QUS T-score ≤ -1,8 sd without considering age.

The accuracy of using the FRAX with a MOF risk ≥ 8.5% for osteoporosis screening (T-score ≤ -1.8 sd) was poor ( Fig. 1 , Fig. 2 , Table 2 and Table 3 ), with an AUC of 0.604 (95% confidence interval [95%CI]: 0.509–0.694) for women under 65 years of age, and of 0.642 (95%CI: 0.571–0.709) when age was not considered. Including MM data in the statistical analysis led to a significant improvement in the group of women under 65 years of age, with an AUC of 0.705 (95%CI: 0.612–0.786).

**Table 2:** Analysis of Figure 1

	Area under the curve	Standard Error	95% Confidence Interval
FRAX MOF	0.604	0.059	0.509–0.694
FRAX MOF + CC	0.705	0.058	0.612–0.786

Abbreviations: CC, circumferences; FRAX, Fracture Risk Assessment Tool; MOF, major osteoporotic fracture.

**Table 3:** Analysis of Figure 2

	AUC	Standard Error	95% CI
FRAX MOF	0.642	0.041	0.571–0.709
FRAX MOF + CC	0.654	0.043	0.583–0.720

Abbreviations: 95%CI, 95% confidence interval; AUC, area under the curve; CC, circumferences; FRAX, Fracture Risk Assessment Tool; MOF, major osteoporotic fracture.

Table 4 shows that the NOGG tool had a sensitivity of 17% to identify individuals with QUS T-score ≤ -1.8 sd, as well as a specificity of 84%, a positive predictive value of 31%, and a negative predictive value of 71%.

**Table 4:** Comparison between medium- or high-risk and low-risk subjects in the NOGG clinical guideline to identify individuals with T-score ≤ -1,8 sd on calcaneal qualitative ultrasound

	T-score ≤ -1,8 n (%)	T-score > -1,8 n (%)	*p* *
Medium- or high-risk on the NOGGclinical guideline	10 (5)	22 (11)	0.7910
Low-risk on the NOGG clinical guideline	48 (24)	118 (60)	

Abbreviation: NOGG, National Osteoporosis Guideline Group.

## Discussion

In the present study, e observed that the ability to identify low bone mass in women under 65 years of age was greater when measurements of the circumferences of the arm, calf, and thigh were associated with the FRAX with a MOF risk ≥ 8.5%.

Although studies are scare, especially regarding the population under 65 years of age, the relationship between MM measurements and and the risk of fracture has already been evaluated. Faulkner et al. 32 analyzed 8,074 women aged 67 years or older during 1,6 years, and found a correlation between the length of the hip axis and increased risk of trochanteric fracture (odds ratio [OR] = 1.6; 95%CI: 1.0–2.4) and femoral neck fracture (OR = 1.9; 95% CI 1.3–3.0). In another study, Farmer et al., 31 who evaluated a population aged between 40 and 77 years, found a relationship of the arm muscle area and the thickness of the triceps skinfold with an increased risk for hip fractures. 12 31 32

It stands out that the absolute risk of fracture for any bone density value among young postmenopausal women is small compared with the risk for those aged over 65 years. According to Doherty et al., 33 the probability of vertebral or hip fracture at 5 years is of 03% and 0% respectively among women aged between 50 and 54 years, of 0.5% and 0.2% among those aged between 55 and 59 years, and of 1% and 0.2% among women aged between 55 and 64 years. The performance of universal screening with the bone densitometry test for all women over 50 years of age is an expensive and ineffective strategy. Accordingly, providing a more accurate screening alternative is critical as a public health strategy.

Currently, although numerous diagnosis tools have already been developed, there is no consensus as to which should be used, or even as to which guideline should be followed to identify low bone mass in young postmenopausal women. In 2014, Crandall et al 12 evaluated the diagnostic tools for this population, and obtained a FRAX AUC value of 0,60, which is considered low, and is similar to that found in the present study (0,604). When comparing it to other screening methods, the authors 12 found that the sensitivity and specificity of the OST, which uses only age and weight, was higher than those of the FRAX with a MOF risk ≥ 8.4%.

The mean of age of 64 years shows that the sample of the present study is representative of young postmenopausal women, the focus of the study, who, as expected, showed better performance, strength and MM. Despite this, 20% of this population presented T-scores ≤ -1.8 sd and 7.8% ≤ -2.5 sd in the QUS. This finding differs from the small fracture rate expected among this population in 5 years, as documented by Doherty et al. 33 in 2001, and it can be explained by the fact that the sample was not chosen at random, but consisted of women who saught medical assistance at a health fair.

The low performance of the NOGG tool when compard with the QUS can be explained by the fact that the majority of the women included in the study was young, with good bone mass levels. Another relevant factor is that only 32 patients, a low number, were classified as high-risk. In any case, there are few studies evaluating the performance of the NOGG tool among the Brazilian population, especially among women aged ≥ 65 years. Even though the QUS is not a standard for the diagnosis of osteoporosis, it presents high clinical applicability in terms of the prediction of fractures, as confirmed by Moayyeri et al. 3 in a study with a follow-up of more than 200 thousand person-years.

The results of the present study are substantial, considering that the sample comprised a significant number of the population, people from the community, and not previously selected, as occurs with patients cared for in outpatient clinics. Nevertheless, the present research may be considered notable due to the fact that it is, perhaps, the first Brazilian study to correlate risk factors for sarcopenia with the diagnosis of osteoporosis or the risk of fracture. The results have a relevant potential for application in the medical practice.

The present study has several limitations. The ethnic groups included present different body fat distribution, a factor considered a bias by Lee et al. 18 during the development of the equation for the MM analysis. Further, the population evaluated was overweight, which interfered with the interpretation of the MM and sarcopenia results. Another fact considered relevant was that the population had anthropometric measurements taken in a non-standardized way in relation to their clothing, because it was an event open to the public, with a high flow of people. In addition, it is known that the QUS, a method used as a parameter to assess bone mass, is not a standard for the diagnosis. However, it is a tool that may be used at a health fair with a reasonable degree of accuracy. The present study will be repeated, with the possibility of inviting patients and applying bone densitometry in the future.

The association of measurements of the calf, arm and thigh improved the accuracy of the FRAX to detect individuals under 65 years of age with lower bone mass on the QUS. This demonstrates the importance of evaluating parameters related to MM in the identification of individuals at risk of developing osteoporosis or incurring in fragility fractures. Associating such measures with the FRAX tool, improving the performance of these strategies, has a great potential regarding osteoporosis care, especially among young postmenopausal women. Further studies are needed to confirm the findings of the present study and establish new approaches in the screening and diagnosis of the risk of fracture due to frailty.

## Conclusion

The association of arm, thigh, and calf measurements increased the accuracy of the FRAX to screen for osteoporosis among women under 65 years of age.
